# Proportion of Successful Lumbar Punctures in Infants Younger Than Three Months

**DOI:** 10.7759/cureus.51507

**Published:** 2024-01-02

**Authors:** Rei Miyake, Kento Ikegawa, Hiroshi Hataya, Yoshihiko Morikawa

**Affiliations:** 1 Department of General Pediatrics, Tokyo Metropolitan Children's Medical Center, Tokyo, JPN; 2 Clinical Research Support Center, Tokyo Metropolitan Children's Medical Center, Tokyo, JPN; 3 Clinical Research Support Center, Tokyo Metropolitan Children’s Medical Center, Tokyo, JPN

**Keywords:** bacterial meningitis, risk factors, febrile infant, lumbar puncture (lp), cerebrospinal fluid (csf)

## Abstract

It is important to perform lumbar punctures (LPs) without a single traumatic tap in infants younger than three months owing to the risk of serious complications. The proportion of LPs in which clear cerebrospinal fluid (CSF) was obtained has been previously reported, but some of the procedures involved a traumatic tap. The present study aimed to identify the proportion of LPs in which clear CSF was obtained without a single traumatic tap and the factors associated with successful LPs in infants younger than three months. This retrospective, observational study included children younger than three months who underwent an LP in the pediatric emergency department between April 2018 and March 2021. The primary outcome was the proportion of successful LPs, defined as LPs obtaining clear CSF without a single traumatic tap. Multiple logistic regression analysis was used to identify factors related to successful LPs. Of 126 eligible patients, 121 were included. Among these, 83 (69%) were in the successful group. No factors significantly associated with successful LPs were found. Larger studies based on an accurate definition of successful LPs, such as that provided by this study, are needed to investigate related factors to increase the rate of successful LPs in this age group.

## Introduction

Lumbar punctures (LPs) are commonly performed in children to assess for bacterial meningitis but are associated with complications, such as post-LP headache, traumatic tap, cranial neuropathies, back pain, nerve root injury, meningitis, and cerebral herniation [1]. Among these, the traumatic tap, which occurs if the needle enters an epidural vein during the puncture, is of particular concern because it occurs frequently (10-30% [2,3]), renders accurate assessment of cerebrospinal fluid (CSF) difficult, and leads to unnecessary antibiotic use, longer hospitalization, and increased health care costs [4]. Unnecessary antibiotic use can in turn lead to higher morbidity and death rates by promoting antimicrobial resistance [5]. It is clinically important not to have even one traumatic tap during an LP even if clear CSF is eventually obtained because complications such as neuropathy of the lower limbs, back pain, headache, visual disturbance, and impaired consciousness have been reported as a result of bleeding into the spinal fluid cavity caused by a traumatic tap [1,6,7]. This is especially the case in infants younger than three months because of the large number of LPs performed in this age group [8] and the risk of severe complications, such as lower limb paralysis caused by damage to the conus medullaris, which is especially vulnerable due to its low position [9].

To enable clear CSF to be obtained without a traumatic tap in infants younger than three months, the proportion of LPs with clear CSF without a traumatic tap, i.e., successful LP, needs first to be established; then the factors associated with its success need to be identified. Although a previous study conducted in an emergency department (ED) reported that the proportion of LPs with clear CSF in infants younger than 60 days was 86% [10], in some of the procedures a traumatic tap occurred before clear CSF was extracted, resulting in an overestimate of the proportion of successful LPs, defined as LPs without a single traumatic tap. Bedetti et al. reported the proportion of successful LPs without a single traumatic tap in infants younger than 90 days in a neonatal intensive care unit (NICU) [11]. However, no previous studies have demonstrated the proportion of successful LPs in the ED setting. The present study aimed to establish the proportion of successful LPs and identify the factors associated with their success in children younger than three months visiting an ED.

## Materials and methods

Study setting

The present, retrospective, observational study was conducted in the ED at Tokyo Metropolitan Children’s Medical Center, a pediatric tertiary care hospital in Japan with an annual ED intake of approximately 35,000 outpatients.

Study population

Infants younger than three months who underwent LPs with the standard palpation method in the ED between April 2018 and March 2021 were eligible for inclusion. The exclusion criterion was refusal to participate. Multiple procedures in the same patient were included individually.

Data collection and analysis

Electronic medical records of infants who were younger than three months when they underwent CSF analysis at the ED were reviewed for relevant data, including patient characteristics (age, sex, weight, gestational age, birth weight, underlying disease, medical indications for LPs, consciousness status), physicians’ years of pediatric clinical experience, procedural factors (timing, local anesthetic use, sedative use), number of attempts, duration of LPs, red blood cells (RBCs) in CSF, types and doses of antibiotic, and CSF culture results.

Standard LP procedure

The standard method of performing an LP in our ED is as follows: the physician places the patient in the lateral decubitus position and selects a lumbar interspace by palpating the Jacoby line, a horizontal line connecting the highest points of both iliac crests, then punctures the site using a 23-gauge needle after disinfection. If the obtained CSF is bloody, the physician removes the needle and performs the procedure again at another lumbar interspace. In this situation, the clinician or nurse notes in the medical records that the CSF was bloody. If the obtained CSF is macroscopically non-traumatic or contains just a little blood, it is sent to a laboratory for a cell count and culture. If it contains a small amount of blood, the RBC is counted to correct the number of white blood cells (WBC) in the CSF using the peripheral RBC to WBC ratio, and the laboratory technician records the results in the laboratory records. If non-traumatic CSF is unable to be obtained after repeated attempts, the procedure is discontinued.

Definition of LP results

**Figure 1:**
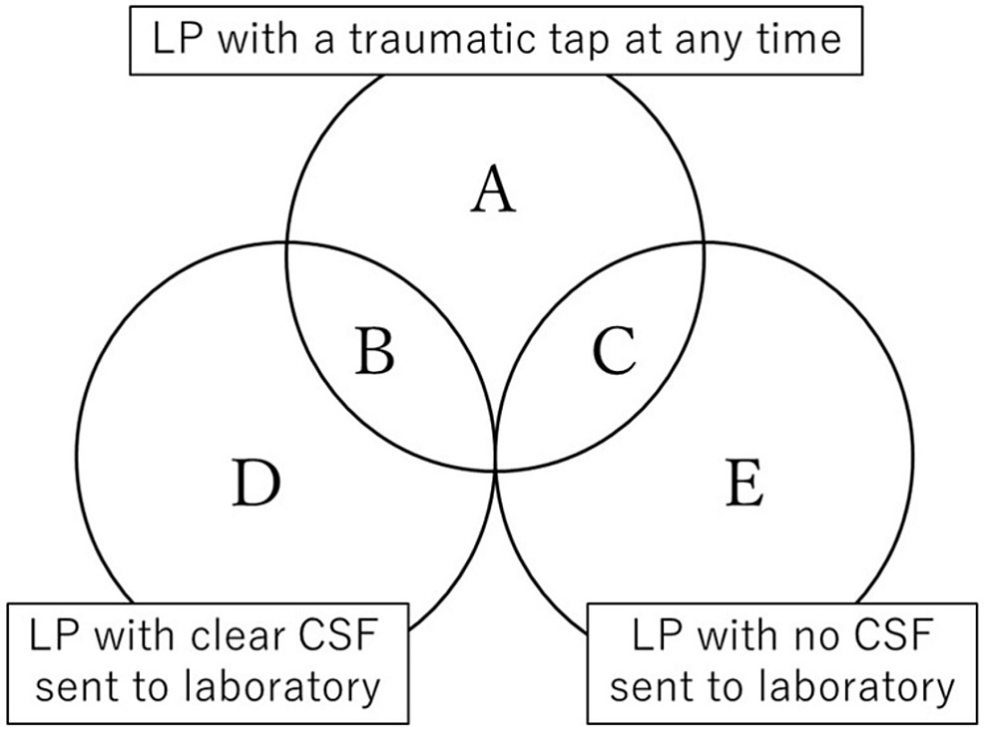
Data classification of LP The LP categories with their definition are A: LP with a traumatic tap during the procedure with RBC ≥ 10,000 /mm^3^ in CSF analyzed by a laboratory; B: LP with a traumatic tap during the procedure with CSF analyzed by a laboratory appearing to be non-traumatic or containing RBC < 10,000 /mm^3^; C: LP with a traumatic tap with no CSF sent to a laboratory for analysis; D: LP without a single traumatic tap with CSF analyzed by a laboratory appearing to be non-traumatic or containing RBC < 10,000 /mm^3^ (successful LP); E: LP obtaining no CSF or blood. LP: lumbar puncture, CSF: cerebrospinal fluid

Figure 1 shows the classification of LPs. An LP was defined as traumatic (categories A, B, and C) if the CSF was determined by the physician to be bloody or laboratory analysis demonstrated RBC ≥ 10,000 /mm^3^. An LP was defined as successful (category D) if clear CSF was collected without a single traumatic tap. Clear CSF was defined as CSF containing no visible blood or having RBC < 10,000 /mm^3^ on laboratory analysis. All other cases were defined as unsuccessful, i.e., involving a traumatic tap or failure to obtain CSF (categories A, B, C, and E). The RBC cutoff of 10,000 /mm^3^ was chosen to reflect a level of blood contamination at which the CSF WBC becomes difficult to determine. If the CSF contains RBC ≥ 10,000 /mm^3^, the WBC increases by more than 10 /mm^3^ according to the CSF RBC: WBC correction ratio of 500:1 to 1000:1 [10]. Since the normal CSF WBC is < 19 /mm^3^ in neonates and < 9 /mm^3 ^in infants younger than three months [12], an increase of CSF WBC > 10 /mm^3^ in a traumatic LP makes measurement of the CSF WBC value difficult.

Definition and classification of variables

Fever was defined as a body temperature of 38℃ or higher. An underlying disease was defined as a disease capable of interfering with the LP procedure, such as a tumor or wound on the back, scoliosis, neuromuscular disease, or chromosomal abnormality. The duration of LPs was defined as the time from needle insertion to needle extraction at the end of the procedure. Consciousness status was classified as A or other than A on the AVPU scale (Alert, Responsive to Voice, Responsive to Pain, Unresponsive) [13]. A patient was considered to have been treated for bacterial meningitis if an antibiotic for bacterial meningitis was administered.

Outcome measures

The primary outcome was the proportion of successful LPs (D in Figure 1). The secondary outcomes were the proportion of 1) traumatic LP (A, B, and C in Figure 1); 2) LP with clear CSF on the first attempt; 3) LP with any CSF regardless of the RBC count (A, B, and D in Figure 1); 4) the difference in potentially related factors, number of LP attempts, and duration of LP between successful and unsuccessful procedures; 5) the difference in treatment after hospitalization between the group with clear CSF (B and D in Figure 1) and the group without clear CSF (A, C, and E in Figure 1); and 6) the relationship between successful LP and the relevant factors.

Statistical analysis

The background characteristics of the patients were expressed using the mean and standard deviation (SD) for continuous variables and percentages for categorical variables. The primary outcome was estimated and described in percentages. The unpaired t-test was used for continuous variables, and the chi-square test or Fisher’s exact test was used for categorical variables to evaluate the differences between successful and unsuccessful LP in terms of potentially relevant factors, the number of attempts and duration of LP, and the differences of treatment after hospitalization between the LP with clear CSF and LP without clear CSF. Multiple logistic regression was carried out to determine the relationship between successful LPs and factors of potential relevance. Two-sided P < 0.05 was considered to indicate statistical significance. All statistical analyses were performed using IBM SPSS Statistics for Windows, Version 27 (Released 2020; IBM Corp., Armonk, New York, United States).

Ethical considerations

The present study was approved by the Ethics Committee of Tokyo Metropolitan Children’s Medical Center (2020b-189) and was performed in accordance with the ethical standards laid down in the Declaration of Helsinki, the Ethical Guidelines for Medical and Health Research Involving Human Subjects of the Ministry of Health, Labour and Welfare of Japan, and the Strengthening the Reporting of OBservational studies in Epidemiology (STROBE) guidelines. The ethical committee waived the need for informed consent. An opt-out clause was provided on the institutional website.

## Results

Patient characteristics

In total, 126 infants younger than three months underwent LPs in the ED between April 2018 and March 2021. Their mean age was 20.9 days (SD, 15.1), and 70 (55.6%) were male (Table 1). One patient had two ED visits for LP. Only one infant had an underlying disease (trisomy 21).

**Table 1:** Patient characteristics SD: Standard deviation, LP: lumber puncture

Characteristics	N = 126
Age, mean (SD), days	20.9 (15.1)
0-28, n (%)	101 (80)
29-60, n (%)	20 (16)
61-89, n (%)	5 (4)
Male sex, n (%)	70 (56)
Weight, mean (SD), kg	3.55 (0.76)
Gestational age, mean (SD), week	38.5 (1.4)
Birth weight, mean (SD), kg	3.02 (0.44)
Underlying disease, n (%)	1 (0.8)
Medical indication for LP, n (%)	
Fever	122 (97)
Convulsion	1 (0.8)
Unconsciousness	1 (0.8)
Other	2 (1.6)

Data classification

In the classification shown in Figure 1, the number of LPs per category was 12, 11, 12, 83, and 3 in categories A, B, C, D, and E, respectively. The remaining five patients were not classified into any of the categories because their CSF RBC was not counted even though the CSF samples were determined to contain blood by laboratory technicians. These five instances were therefore excluded from the outcome analysis.

Main results

Of the 121 patients, 83 (D, 69%) were in the successful LP group, and 35 (A+B+C, 29%) were in the traumatic LP group. Seventy-two LPs (60%) had clear CSF on the first attempt, and 106 (A+B+D, 88%) had CSF regardless of RBCs or the number of LP attempts. The mean (SD) number of LP attempts was 1.2 (0.7) in the successful LP group and 3.7 (2.2) in the unsuccessful LP group (p < 0.001). The mean (SD) procedural duration in the successful LP group was significantly shorter than in the unsuccessful LP group (p < 0.001) at 7.3 (6.0) minutes and 19.6 (14.6) minutes, respectively (Table 2).

**Table 2:** Comparison of background factors and results between the successful LP and unsuccessful LP SD: Standard deviation, LP: lumber puncture AVPU: Alert, Responsive to Voice, Responsive to Pain, Unresponsive

	Successful LP N = 83	Unsuccessful LP N = 38	P-value
Background factors			
Age, mean (SD), days	22.1 (15.1)	18.2 (15.4)	0.189
Weight, mean (SD), kg	3.6 (0.7)	3.5(0.9)	0.912
Physicians’ years of pediatric clinical experience, n (%)			0.794
< 1	26 (31)	10 (26)	
1-3	31 (37)	14 (37)	
> 3	26 (31)	14 (37)	
Local anesthetic use, n (%)	60 (72)	29 (76)	0.641
Sedative use, n (%)	3 (3.6)	1 (2.6)	1.00
A of AVPU, n (%)	79 (95)	36 (95)	1.00
Time at LP performance, n (%)			0.646
9 am - 5 pm	32 (39)	13 (34)	
5 pm - 9 am	51 (61)	25 (66)	
Results			
No. of attempts, mean (SD)	1.2 (0.7)	3.7 (2.2)	< 0.001***
Duration of LP, mean (SD), min.	7.3 (6.0)	19.6 (14.6)	< 0.001***

Factors associated with LP success

Table 2 shows the unadjusted relationship between each potentially relevant factor and LP success. Univariate analysis indicated that no factors were significantly associated with LP success. Multivariate analysis demonstrated no statistically significant relationship between LP success and the factors of interest (age, physician’s experience, local anesthetic use) (Table 3).

**Table 3:** Multivariable logistic regression analysis of successful LPs LP: Lumbar puncture, CI: confidence interval

Factors	Successful LP Odds ratio (95% CI)
Age	0.7 (0.2-1.8)
Physician's years of pediatric clinical experience	
< 1	1 [Reference]
1-3	1.4 (0.5-3.7)
> 3	1.1 (0.4-2.8)
Local anesthetic use	1.3 (0.5-3.2)

Clinical course

The proportion of patients receiving antibiotics for suspected bacterial meningitis was 31% (29/94) in the group with clear CSF and 52% (14/27) in the group without clear CSF (p = 0.044). Most of these patients (40/43) received antibiotics for only 48 hours while the remaining three patients completed their treatment for bacterial meningitis caused by Streptococcus agalactiae. A CSF culture of two of these patients, both in the successful LP group, grew S. agalactiae. The remaining patient, who was in the traumatic LP group, was also treated for bacterial meningitis despite having a negative CSF culture result after a blood culture grew S. agalactiae, raising the possibility that the pathogen may have been iatrogenically introduced into the CSF via the traumatic tap.

## Discussion

The present study found that the proportion of successful LPs was 69% in infants younger than three months and that there were no factors significantly related to LP success. A unique feature of this study was that the successful LP group included only procedures without a traumatic tap. We focused on whether the LP involved a traumatic tap rather than only on whether clear CSF was eventually obtained in order to account for the complications of the traumatic tap.

The proportion of successful LPs was lower than in a previous study which reported a figure of 86% [10]. Two differences in the definitions of LP success in the respective studies may account for the discrepancy in these results. First, our study included all LPs, whereas the previous report included only LPs with a verifiable cell count, which means the previous study’s definition excluded from the denominator LP which failed to obtain CSF. Second, the present study defined a successful LP as one that obtained clear CSF without a single traumatic tap (D in Figure 1) while the previous study defined it as any LP obtaining clear CSF (B or D in Figure 1). The latter study considered an LP to be successful even if a traumatic tap occurred as long as clear CSF was obtained for laboratory analysis, thus producing a higher proportion of successful LPs. If the definition of the previous study had been used in the present study, the proportion of successful LPs would have been 89%, in line with the figure reported in the previous study.

Our study further found that the proportion of traumatic LPs was 29%, which was higher than in the figure reported by the previous study (14%) [10]. This discrepancy may also have been caused by the different definitions of traumatic LPs used. A traumatic LP was defined in our study as an LP with CSF containing RBC ≥ 10,000 /mm^3^ or judged to be bloody by the clinician at any time during the procedure (A, B, and C in Figure 1). On the other hand, the previous study defined a traumatic LP as an LP with CSF containing RBC ≥ 10,000 /mm^3^ based on laboratory analysis (A in Figure 1). The latter definition did not include LPs with a traumatic tap as long as clear CSF was eventually obtained for laboratory analysis. If the present study had employed this definition, the proportion of traumatic LPs would have been 11%, in line with the figure reported in the previous study.

Our definition of successful LPs marks an improvement over the previous definition for two reasons: first, our definition allowed a more accurate calculation of the proportion of successful attempts by including all the LPs in the denominator; second, it excluded from the successful LP group procedures involving a traumatic tap at any time during an LP because even one traumatic tap raises the risk of complications, such as intraspinal hemorrhage, neuropathy, and iatrogenic meningitis, as seen in the case of bacteremia in the present study. Bedetti et al. reported that 62% of LPs in infants younger than 90 days in the NICU [11] were successful without any traumatic tap, which agrees with our finding despite their study being conducted in the NICU rather than the ED.

The number of attempts and duration of LPs decreased to a significantly greater degree in the successful LP group than in the unsuccessful LP group. A successful LP may reduce the patient’s physical and mental discomfort [14,15], and a smaller number of punctures may also lower the risk of complications.

Our study found no factors significantly associated with successful LPs in infants younger than three months. This result was the same regardless of the RBC count in the five LPs for which the CSF RBC count was not verified. Although previous reports have shown that older age was related to LP success in this age group [10,16], the present study found no significant association between age and LP success. This finding may be due to the relatively smaller population in our study than in the previous studies. Daytime, physician’s experience, and local anesthetic use, which are reportedly related to LP success in patients younger than 18 years of age [17,18], were also not obviously associated with LP success in our study.

The proportion of patients who received treatment for bacterial meningitis was smaller in the group with clear CSF than in the group without clear CSF although the actual rate of bacterial meningitis was small. This finding also underscores the importance of obtaining clear CSF to decrease the hospitalization rate, reduce healthcare costs, and prevent antimicrobial resistance by avoiding high-dose antibiotic therapy [4].

The present study has several limitations. First, because it was retrospective, there may have been a measurement bias. For example, some factors reportedly related to LP success, such as the patients’ movements and use of a spinal needle with the stylet in, were unable to be verified. Second, the CSF RBC count was determined only when the CSF sample sent to a laboratory appeared to contain blood, which may have led to an inaccurate assessment of traumatic LPs. LPs with CSF RBC < 10,000 /mm^3^ may have been mistakenly categorized into the traumatic LP group when the clinician macroscopically judged the CSF to be bloody without laboratory analysis. Third, the characteristics of our hospital may have affected the proportion of successful LPs. Pediatric residents had more opportunities to perform LP than experienced physicians at our hospital. However, our study demonstrated no significant association between LP success and physician's experience. Fourth, we did not perform the sample size calculation before conducting the study.

## Conclusions

The proportion of successful LPs as per our definition in infants younger than three months was 69%. Our definition could be more appropriate than that used in the previous study because all instances of LPs performed in the cohort were included, and LPs with a traumatic tap were not included in the successful LP group in view of the complications associated with traumatic taps. Performing a successful LP is important also because it decreases the number of punctures and the procedural duration while reducing high-dose antibiotic therapy. No factors associated with LP success in infants younger than three months were able to be identified. Prospective studies with a larger population using our definition of successful LPs are needed to investigate relevant factors that might be manipulated to increase the LP success rate in patients in this age group.
